# Practical 3-D Beam Pattern Based Channel Modeling for Multi-Polarized Massive MIMO Systems †

**DOI:** 10.3390/s18041186

**Published:** 2018-04-12

**Authors:** Saeid Aghaeinezhadfirouzja, Hui Liu, Ali Balador

**Affiliations:** 1 Department of Electronics Engineering, Shanghai Jiao Tong University, Shanghai 200240, China; s.aghaei@sjtu.edu.cn (S.A.); huiliu@sjtu.edu.cn (H.L.); 2 Department of Innovation, Design and Technology (IDT), Mälardalen University, 72123 Västerås, Sweden; 3 RISE SICS Västerås, Stora gatan 36, 722 12 Västerås, Sweden

**Keywords:** 3-D massive MIMO channel modeling, antenna elements space, elevation angle of departure, elevation angle of arrival, azimuth angle of departure, azimuth angle of arrival

## Abstract

In this paper, a practical non-stationary three-dimensional (3-D) channel models for massive multiple-input multiple-output (MIMO) systems, considering beam patterns for different antenna elements, is proposed. The beam patterns using dipole antenna elements with different phase excitation toward the different direction of travels (DoTs) contributes various correlation weights for rays related towards/from the cluster, thus providing different elevation angle of arrivals (EAoAs) and elevation angle of departures (EAoDs) for each antenna element. These include the movements of the user that makes our channel to be a non-stationary model of clusters at the receiver (RX) on both the time and array axes. In addition, their impacts on 3-D massive MIMO channels are investigated via statistical properties including received spatial correlation. Additionally, the impact of elevation/azimuth angles of arrival on received spatial correlation is discussed. Furthermore, experimental validation of the proposed 3-D channel models on azimuth and elevation angles of the polarized antenna are specifically evaluated and compared through simulations. The proposed 3-D generic models are verified using relevant measurement data.

## 1. Introduction

Massive multiple-input multiple-output (MIMO) technology has gained lots of attention over the past decade since it provides improved link reliability and high system capacity without extra spectral resource. Towards the emergence of the fifth-generation wireless communication system, massive MIMO systems equipped with tens or hundreds of antennas have emerged to meet the increasing data-rate and high spectrum efficiency. In [1,2,3] massive MIMO systems with the increased number of the antenna was proven to give many benefits and advantages, such as increased channel capacity and reduced cost of implementation.

In designing and evaluating massive MIMO systems, it is necessary to have an accurate and efficient massive MIMO channel model. Recently, several actions had been proposed which gave way to important channel models both for MIMO and massive MIMO. A lot of models classified more 2-D channels with a smaller number of antennas, such as [4], without considering the impact of antenna array on channel modeling. Many researchers have chosen the geometry-based stochastic model (GBSM), known as the ellipse model and one/two ring model in [5,6], where the scatters were distributed in regular shapes. Thus, the geometrical relationship between the scatter and the receiver (RX) determines the channel impulse response of these channel models. Very recently, the authors in [7] conducted a survey about MIMO on channel propagation models and signal processing single-cell/single-user. Although, in [8], a non-stationary model for massive MIMO communication systems was proposed; the work was based on a 2-D non-stationary multi-confocal ellipse model.

In [9,10] the works were based on the 3-D theoretical channel model. The authors assumed an infinite number of effective scattered with resulting infinite complexity that can hardly be put into practical use. The authors in [11,12,13] analyzed time-variant geometric properties such as angle of arrivals (AoA) and angle of departures (AoD), taking into account non-stationeries on the time axis. In [11], for an instance, they analyze shortcomings of a selected spatial channel model standard with respect to the identified requirements from other WINNER Work Packages. In [12], the model enables the simulation of the pure propagation channel behavior and the inclusion of the effects of different antennas and access schemes. In [13], the intra-path delay spread is considered which is the distance between user equipment (UE) and the last bounce scatter of each path for time-variant phases.

A 3-D non-stationary twin-cluster channel model was proposed in [14] for massive MIMO systems considering their variation on both time and array axes. However, the impact of the cluster’s appearance and disappearance in the elevation angles was only capable of a near-field condition based on the spherical wavefront. In [15], the author investigated antenna configuration and polarization for MIMO channels, without considering correlation at the antenna arrays for massive MIMO. In addition, in [16], the authors considered polarized antenna without investigating the antenna element spacing. Furthermore, the work in [17] was based on the multi-polarized fixed wireless channel based on 2-D omnidirectional antenna elements.

To further enhance performance, the actual trend is to exploit the channel’s degree of freedom in the elevation direction [18]. However, according to measurements observed in [19,20,21,22], the above mentioned channel models [1,2,3,4,5,6,7,8,9,10,11,12,13,14,15,16,17] are not sufficient accurate to capture certain characteristics of massive MIMO channels. Generally speaking, previous contributions on channel modeling have been surveying many subjects without tackling the capture of characteristics of 3-D massive MIMO channels in the far-field. Based on these references, we shed light on the current technique to develop a clearer understanding of the movement direction of the antenna array, space between antenna elements and cluster movements. Prior to this, one of the reasonable ways to extract an additional degree of 3-D massive MIMO channels in the far-field is to adjust the beam pattern in the vertical direction for each individual user to improve the signal strength at the receiver.

To improve the 3-D massive MIMO for the far-field effects, three important aspects should be considered to provide an accurate channel modeling in practice.

**First**, nowadays, 3-D channel models show that spherical/plane wave-fronts cannot fulfill the elevation angles with an increased number of antenna elements at a wider space, especially when the user is in the same azimuth angle but at a different elevation. The 3-D beam pattern which is known to provide higher elevation angle is therefore proposed instead of spherical wavefronts. Based on the distance between transmitter (TX) and RX and increased dimension of antenna, spherical waves are emitted in a spherical shape and are considered as a plane wavefront. So, this paper contributes a phenomenon to investigate the beam waves to provide higher elevation angle is therefore proposed instead of spherical/plane wavefronts. Considering this fact, a 3-D massive MIMO in the far-field is assumed such that the phase of each antenna element is determined by the geometrical relationships and EAoAs and EAoDs on the antenna arrays are equal to each antenna element with same power angular spread (PAS). This fact is only possible in the theoretical view if we assumed that the wavefront for each wireless link is spherical. Therefore, in this paper, beam patterns with different PAS were considered to provide various phase excitations for different EAoAs toward different directions of travels (DoTs) on the antenna array with the higher degree of freedom on both the TX and RX.

**Figure 1:**
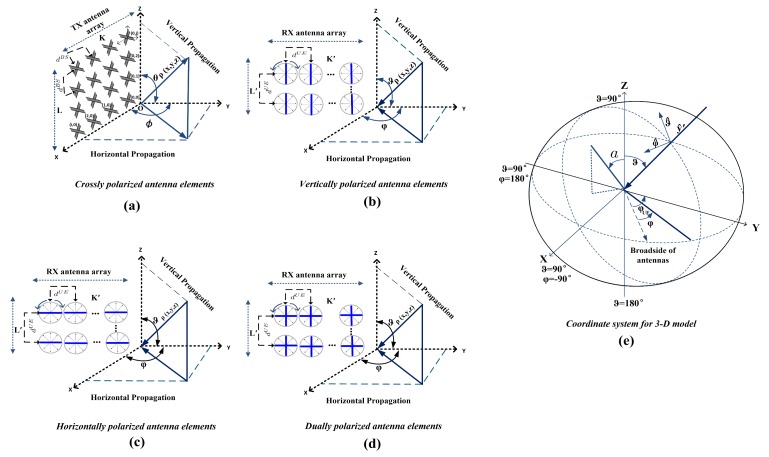
Detailed of antenna configuration X${}_{Pol}$, V, H, and V/H polarizations of an antenna elements in the horizontal and vertical angles.

**Second**, in the massive MIMO antenna, an increase in the dimension of antenna array results in the high correlation between the antenna elements and inaccuracy of channel coefficients. Therefore, for the sake of massive MIMO system design and performance evaluation, it is indispensable to investigate the correlation between antenna elements. This leads us to utilize the spaced rectangular antenna array (SRAA) and spaced uniform linear array (SULA) on received spatial correlation (RSC), considering various elevation angle of arrivals (EAoAs) and azimuth angles of arrivals (AAoAs) for the different antenna elements. This technique provides more accuracy in generating the channel coefficients for a massive MIMO system, where all the antenna elements need to be addressed uniquely at the antenna array to recognize their paths from the elements to the clusters. Antenna element spaces (AESs) technique is an inter-element spacing where the SRAA and SULA are divided into the number of antenna elements in the horizontal and elevation direction of the dipole and omnidirectional antennas. This technique can also be used for any arbitrary choice of the antenna pattern and distribution of azimuth and elevation angles for the polarized massive MIMO antenna with the different configuration of vertical (V), horizontal (H), and Dual (V/H) polarizations as shown in Figure 1.

**Third**, the appearance of clusters on the antenna array is another important characteristic of massive MIMO channel models. In conventional massive MIMO channel models, it is assumed that a cluster is always observable to the entire antennas elements. This model, on the other hand, proposes a non-stationary model based on the movement of the user where the cluster may appear as at least one antenna element and its adjacent elements.

In this paper, we first give a comprehensive survey of the antenna campaigns conducted in different polarization patterns for different scenarios and address the recent advances of the LTE antenna pattern. Then, we propose a framework for deriving the statistical properties of these channels. This channel model is developed aiming to capture the far-field effects with beam pattern antenna arrays and non-stationary properties of clusters on both the time and array axes. Scenarios have a close relationship with channel modeling and measurements.

Note that the measured data is based on the LTE specification as shown in Table 1, such as: using duplexing schemes as time division duplex (TDD) and frequency division duplex (FDD), with 10 (MHz) channel bandwidth, 1024 Sub-carriers, 80, 72 normal cyclic prefix length, 140 symbols, 50 Resource blocks for transmission block configuration, quadrature phase-shift keying (QPSK), 16-quadrature amplitude modulation (QAM) modulation schemes and orthogonal frequency-division multiplexing (OFDM) for multiple access schemes.

The major contributions of this paper are summarized as follows:

- The impact of beam patterns has been proposed for 3-D massive MIMO channel model for different dipole and omnidirectional antenna elements. Therefore, the beam pattern provide different phase excitation towards different DoTs in the far-field. Given that, it also provides various AoDs and AoAs for each antenna element, contributing different correlation weights for rays related towards/from the clusters. As far as the author’s knowledge is concerned, a practical 3-D channel model for massive MIMO using beam pattern assumption in the far-field has not been considered, yet.
- A closed-form expression for AES has also been studied to reduce the RSC in the horizontal and elevation directions of the antenna array that can be accurately represented as an important aspect of a polarized antenna in 3-D space. Therefore, to design and evaluate a massive MIMO system, the investigation of correlations between antenna elements are necessary. This fact is possible in utilizing the SRAA and SULA, where all the antenna elements need to be addressed uniquely at the antenna array for investigating received spatial correlation. In fact, the model is providing an accurate observation to investigate the received spatial correlation based on the antenna polarizations.
- The movement of the user and clusters make our channel non-stationary which is applied to both time and array axes. It means that the behavior of the clusters varies at different times of EAoAs and AAoAs. Therefore, receiving clusters are observed to at least one antenna element and its adjacent elements depend upon their distance to the clusters at the RX. A novel cluster evolution algorithm in the system level is developed in the antenna pattern.
- The impact of the 3-D beam pattern channel model and elevation angle of the aforementioned channel properties is being investigated by comparing it with those of the 3-D conventional channel model. Statistical properties of the proposed massive MIMO channel model such as ECC and RSC, including signal-to-noise ratio (SNR) of the non-stationary channel model, were investigated. The proposed model has been valid for the far-field effects on the massive MIMO scenarios at the cell edge and the result looks convincing. This might provide a more accurate model for the current LTE-A system. Our good implementation models substantially facilitate the implementation of further techniques for different modeling, especially for massive MIMO antenna where the antenna space will affect the MIMO performance.

This paper is organized as follows: Section 2 proposes the extension of 3-D massive MIMO antenna pattern considering both a 3-D beam pattern and the spaces between elements. This includes geometrical properties derived under the 3-D beam pattern as well as the AES technique which describes different configurations on the array axis, in practice. Section 3 presents the steps and methodology in generating a complete 3-D beam pattern channel model. In addition, the received spatial correlation in detail for massive MIMO systems is contained in Section 4. Furthermore, Section 5 shows the simulation results and conclusions are finally drawn in Section 6.

## 2. 3-D Antenna Configuration

In this section, 3-D massive MIMO antenna system contributes beam pattern being proposed, where the type of antenna polarization decides the pattern of the beam and polarization at the TX and RX. In addition, the AES is also localizing each antenna element in the horizontal and vertical direction with different polarizations. Prior to this, the channel’s elevation degree of freedom in vertical angle can be more exploited and the transmit power can be more concentrated heading to the user.

### 2.1. 3-D AES and Antenna Element’s Positioning

Let us now consider an LK transmit antenna which is composed of l=0,…L−1 and k=0,…,K−1 identical antenna elements, which is indexed by $l_{th}$ row and $k_{th}$ column, respectively. Similarly, $L^{\prime }K^{\prime }$ receive antenna which is composed of $l^{\prime }=0,\ldots ,L^{\prime }-1$ and $k^{\prime }=0,\ldots ,K^{\prime }-1$ identical antenna elements, where index by $l_{th}^{\prime }$ row and $k_{th}^{\prime }$ column. The outgoing/incoming wave directions are fixed along *y* axis and the antenna arrays are fixed at *x* and *z* axes both for TX and RX. Prior to this, $\left(\theta_{EAoD},\phi_{AAoD}\right)$ and $\left(\vartheta_{EAoA},\varphi_{AAoA}\right)$ are the elevation and azimuth angles for TX and RX, respectively. In the SRAA at the base station (BS) and SULA, the minimal distance between antenna elements denote as $d^{BS}$ and $d^{UE}$, respectively. In Figure 1a, lk is a point of (x,y,z) of the distance above the origin *O* from an array at the (x,z) axes and the distance vector between the TX and RX is D=(D,0,0). The calculation of the antenna element’s positioning, considering different antenna polarizations, can be expressed as follow sub-sections.

#### 2.1.1. Transmit Antenna Configuration

Massive MIMO with dipole antennas are crossly implemented at the TX. The 3-D Cartesian coordinate systems $\rho_{mn}$ with $X_{m,n}=\left(x_{m,n},y_{m,n},z_{m,n}\right)$ spherical coordinate of a point, where $x_{m,n}=\rho_{m,n}\sin\phi_{AAoD}\cos\theta_{EAoD},y_{m,n}=\rho_{m,n}\sin\phi_{AAoD}\sin\theta_{EAoD}$, and $z_{m,n}=\rho_{m,n}\cos\theta_{EAoD}$. However, antenna element’s point of SRAA can be obtained from its Cartesian coordinates (x,y,z) by the formula

(1) $$\rho_{m,n}=\sqrt{x_{m,n}^{2}+y_{m,n}^{2}+z_{m,n}^{2}}$$

The position of two individual antenna elements, one indexed by lk and the other one indexed by (mn) at the TX can be computed as

(2) $$d_{X_{Pol}}^{BS}=\sqrt{{\left(x_{mn}-x_{lk}\right)}^{2}+{\left(y_{mn}-y_{lk}\right)}^{2}+{\left(z_{mn}-z_{lk}\right)}^{2}}$$

#### 2.1.2. Receive Antenna Configuration

Similarly, in the user side, there are three types of antenna configurations using omnidirectional antenna with V, H, and V/H polarizations for SULA. The 3-D Cartesian coordinate systems $\rho_{m^{\prime },n^{\prime }}$ with $Y_{m^{\prime },n^{\prime }}=\left(x_{m^{\prime },n^{\prime }},y_{m^{\prime },n^{\prime }},z_{m^{\prime },n^{\prime }}\right)$ spherical coordinates of a points, where $x_{m^{\prime },n^{\prime }}=\rho_{m^{\prime },n^{\prime }}\sin\varphi_{AAoA}\cos\vartheta_{EAoA}$, $y_{m^{\prime },n^{\prime }}=\rho_{m^{\prime },n^{\prime }}\sin\varphi_{AAoA}\sin\vartheta_{EAoA}$, and $z_{m^{\prime },n^{\prime }}=\rho_{m^{\prime },n^{\prime }}\cos\vartheta_{EAoA}$. Therefore, antenna element’s point of SULA for V polarization can be obtained from its Cartesian coordinates (x,y,z) by

(3) $$\rho_{m^{\prime },n^{\prime }}^{V}=\sqrt{0+y_{m^{\prime },n^{\prime }}^{2}+z_{m^{\prime },n^{\prime }}^{2}}$$

The position of two vertical antenna elements, one indexed by $l^{\prime }k^{\prime }$ and the other one denote by $\left(m^{\prime }n^{\prime }\right)$ at the RX can be computed as

(4) $$d_{V}^{UE}=\sqrt{0+{\left(y_{m^{\prime }n^{\prime }}-y_{l^{\prime }k^{\prime }}\right)}^{2}+{\left(z_{m^{\prime }n^{\prime }}-z_{l^{\prime }k^{\prime }}\right)}^{2}}$$

In addition, antenna element’s point of SULA for H polarization can be obtained from its Cartesian coordinates (x,y,z) by

(5) $$\rho_{m^{\prime }n^{\prime }}^{H}=\sqrt{x_{m^{\prime }n^{\prime }}^{2}+y_{m^{\prime }n^{\prime }}^{2}+0}$$

The position of two horizontal antenna elements, one indexed by $l^{\prime }k^{\prime }$ and the other one indexed by $\left(m^{\prime }n^{\prime }\right)$ at the RX can be computed as

(6) $$d_{H}^{UE}=\sqrt{{\left(x_{m^{\prime }n^{\prime }}-x_{l^{\prime }k^{\prime }}\right)}^{2}+{\left(y_{m^{\prime }n^{\prime }}-y_{l^{\prime }k^{\prime }}\right)}^{2}+0}$$

Furthermore, antenna element’s point of SULA for V/H polarization can be obtained from its Cartesian coordinates (x,y,z) by

(7) $$\begin{array}{c} \rho_{m^{\prime }n^{\prime }}^{V/H}=\sqrt{x_{m^{\prime }n^{\prime }}^{2}+y_{m^{\prime }n^{\prime }}^{2}+z_{m^{\prime }n^{\prime }}^{2}} \end{array}$$

The position of two vertical/horizontal antenna elements, one indexed by $l^{\prime }k^{\prime }$ and the other one indexed by $\left(m^{\prime }n^{\prime }\right)$ at the RX can be computed as

(8) $$\begin{array}{c} d_{V/H}^{UE}=\sqrt{{\left(x_{m^{\prime }n^{\prime }}-x_{l^{\prime }k^{\prime }}\right)}^{2}+{\left(y_{m^{\prime }n^{\prime }}-y_{l^{\prime }k^{\prime }}\right)}^{2}+{\left(z_{m^{\prime }n^{\prime }}-z_{l^{\prime }k^{\prime }}\right)}^{2}} \end{array}$$

The polarization vector at angle *a* from the *z* axis in Figure 1e, has vertical and horizontal components of the antenna pattern, by expressing the response vector χ in its ϑ and φ components [4] which are proportional to

(9) $$\chi_{l^{\prime }k^{\prime }}^{UE}=\left[\begin{array}{c} \chi_{l^{\prime }k^{\prime }}^{\vartheta }\\ \chi_{l^{\prime }k^{\prime }}^{\varphi } \end{array}\right]=\left[\begin{array}{c} \cos a\sin\vartheta +\cos a\cos\vartheta \cos\varphi \\ \sin a\cos\phi \end{array}\right]$$

where $\chi^{\vartheta }$ and $\chi^{\varphi }$ are the ϑ and φ polarized responses of the antenna at the wave direction of [sinφcosϑ,sinφsinϑ,cosϑ] for the incoming wave in the response of the antenna.

To elaborate the steps and methodology in generating a complete 3-D channel model for massive MIMO systems, considering the beam pattern for different polarizations. The channel impulse response between the $lk_{th}$ and the $l^{\prime }k_{th}^{\prime }$ antenna element both for line-of-sight (LOS) and Non-line-of-sight (NLOS) can be presented as

(10) $$\begin{array}{c} {\left[H\right]}_{lk,l^{\prime }k^{\prime }}\left(t,\tau \right)=\alpha_{LOS}h_{LOS,lk,l^{\prime }k^{\prime }}\left(t\right)+\sum_{i=1}^{I}\alpha_{i}h_{i,lk,l^{\prime }k^{\prime }}\left(t,\tau \right) \end{array}$$

where α is the complex amplitude of the LOS path of $h_{LOS,lk,l^{\prime }k^{\prime }}\left(t\right)$, and NLOS components, path *i*, where i=1,…,I of $h_{i,lk,l^{\prime }k^{\prime }}\left(t,\tau \right)$.

Based on the Equation (10), the effective 3-D radio channel can be derived as [18]

(11) $$\begin{array}{c} {\left[H\right]}_{lk,l^{\prime }k^{\prime }}\left(t,\tau \right)=\alpha_{LOS}\sqrt{gt(\phi_{LOS},\theta_{LOS},\theta^{tilt})}\sqrt{gr(\varphi_{LOS},\vartheta_{LOS},\vartheta^{tilt})}{\vec{a}}_{l^{\prime }k^{\prime }}\left(\varphi_{LOS},\vartheta_{LOS}\right)\times \\ {\vec{a}}_{lk}\left(\phi_{LOS},\theta_{LOS}\right)+\sum_{i=1}^{I}\alpha_{i}\left(t,\tau \right)\sqrt{gt(\phi_{i},\theta_{i},\theta^{tilt})}\sqrt{gr(\varphi_{i},\vartheta_{i},\vartheta^{tilt})}{\vec{a}}_{l^{\prime }k^{\prime }}\left(\varphi_{i},\vartheta_{i}\right)\times {\vec{a}}_{lk}\left(\phi_{i},\theta_{i}\right) \end{array}$$

where (ϕ, θ) are the azimuth and elevation AoD and (φ, ϑ) are the azimuth and elevation AoA and $\theta^{tilt},\vartheta^{tilt}$ are the elevation of the antenna boresight for both TX and RX, respectively. According to the 3-D antenna beam pattern and considering the signal direction of ϕ, θ and φ, ϑ on the antenna arrays, the global patterns for the NLOS components can be represent as $\sqrt{gt(\phi_{i},\theta_{i},\theta^{tilt})}\approx gt_{H}^{BS}\left(\phi_{i}\right),gt_{V}^{BS}\left(\theta_{i},\theta^{tilt}\right)$ and $\sqrt{gr(\varphi_{i},\vartheta_{i},\vartheta^{tilt})}\approx gr_{H}^{UE}\left(\varphi_{i}\right),gr_{V}^{UE}\left(\vartheta_{i},\vartheta^{tilt}\right)$ for the $i_{th}$ path of horizontal and vertical pattern at the TX and RX, respectively. In addition,

(11.1) $$\begin{array}{cc} {\left[H\right]}_{lk,l^{\prime }k^{\prime }}^{V}\left(t,\tau \right)= & \underset{LOS}{\underset{︸}{\alpha_{LOS}\sqrt{gt(\phi_{LOS},\theta_{LOS},\theta^{tilt})}\times {\vec{a}}_{lk}{\left(\phi ,\theta \right)}_{X_{Pol}}\sqrt{gr(\varphi_{LOS},\vartheta_{LOS},\vartheta^{tilt})}\times {\vec{a}}_{l^{\prime }k^{\prime }}{\left(\varphi ,\vartheta \right)}_{V}}}+\\ & \underset{NLOS}{\underset{︸}{\sum_{i=1}^{I}\alpha_{i}\left(t,\tau \right)\sqrt{gt(\phi_{i},\theta_{i},\theta^{tilt})}\times {\vec{a}}_{lk}{\left(\phi ,\theta \right)}_{X_{Pol}}\sqrt{gr(\varphi_{i},\vartheta_{i},\vartheta^{tilt})}\times {\vec{a}}_{l^{\prime }k^{\prime }}{\left(\varphi ,\vartheta \right)}_{V}}} \end{array}$$

(11.2) $$\begin{array}{cc} {\left[H\right]}_{lk,l^{\prime }k^{\prime }}^{H}\left(t,\tau \right)= & \underset{LOS}{\underset{︸}{\alpha_{LOS}\sqrt{gt(\phi_{LOS},\theta_{LOS},\theta^{tilt})}\times {\vec{a}}_{lk}{\left(\phi ,\theta \right)}_{X_{Pol}}\sqrt{gr(\varphi_{LOS},\vartheta_{LOS},\vartheta^{tilt})}\times {\vec{a}}_{l^{\prime }k^{\prime }}{\left(\varphi ,\vartheta \right)}_{H}}}+\\ & \underset{NLOS}{\underset{︸}{\sum_{i=1}^{I}\alpha_{i}\left(t,\tau \right)\sqrt{gt(\phi_{i},\theta_{i},\theta^{tilt})}\times {\vec{a}}_{lk}{\left(\phi ,\theta \right)}_{X_{Pol}}\sqrt{gr(\varphi_{i},\vartheta_{i},\vartheta^{tilt})}\times {\vec{a}}_{l^{\prime }k^{\prime }}{\left(\varphi ,\vartheta \right)}_{H}}} \end{array}$$

(11.3) $$\begin{array}{cc} {\left[H\right]}_{lk,l^{\prime }k^{\prime }}^{V/H}\left(t,\tau \right)= & \underset{LOS}{\underset{︸}{\alpha_{LOS}\sqrt{gt(\phi_{LOS},\theta_{LOS},\theta^{tilt})}\times {\vec{a}}_{lk}{\left(\phi ,\theta \right)}_{X_{Pol}}\sqrt{gr(\varphi_{LOS},\vartheta_{LOS},\vartheta^{tilt})}\times {\vec{a}}_{l^{\prime }k^{\prime }}{\left(\varphi ,\vartheta \right)}_{V/H}}}+\\ & \underset{NLOS}{\underset{︸}{\sum_{i=1}^{I}\alpha_{i}\left(t,\tau \right)\sqrt{gt(\phi_{i},\theta_{i},\theta^{tilt})}\times {\vec{a}}_{lk}{\left(\phi ,\theta \right)}_{X_{Pol}}\sqrt{gr(\varphi_{i},\vartheta_{i},\vartheta^{tilt})}\times {\vec{a}}_{l^{\prime }k^{\prime }}{\left(\varphi ,\vartheta \right)}_{V/H}}} \end{array}$$

in similar way, the 3-D antenna beam pattern on the antenna arrays, the global patterns for the LOS component can be represent as $\sqrt{gt(\phi_{LOS},\theta_{LOS},\theta^{tilt})}$≈$gt_{H}^{BS}\left(\phi_{LOS}\right),gt_{V}^{BS}\left(\theta_{LOS},\theta^{tilt}\right)$ and $\sqrt{gr(\varphi_{LOS},\vartheta_{LOS},\vartheta^{tilt})}\approx gr_{H}^{UE}\left(\varphi_{LOS}\right),gr_{V}^{UE}\left(\vartheta_{LOS},\vartheta^{tilt}\right)$ for the horizontal and vertical pattern at the TX and RX, respectively. Furthermore, vector ${\vec{a}}_{lk}\left(\phi_{i},\theta_{i}\right),{\vec{a}}_{l^{\prime }k^{\prime }}\left(\varphi_{i},\vartheta_{i}\right)$ and ${\vec{a}}_{lk}\left(\phi_{LOS},\theta_{LOS}\right)$, ${\vec{a}}_{l^{\prime }k^{\prime }}\left(\varphi_{LOS},\vartheta_{LOS}\right)$ are the array responses of the TX and RX antennas for LOS component and NLOS components, respectively, by expressing the response vector χ of X${}_{Pol}$, V, H and V/H antenna polarizations in Equations (2), (4), (6) and (8) whose entries are given by

(12) $$\begin{array}{c} {\vec{a}}_{lk}{\left(\phi ,\theta \right)}_{X_{Pol}}=\left[\begin{array}{c} \chi_{lk}^{\theta }\chi_{lk}^{\phi } \end{array}\right]\exp\left(js_{lk}\left(lk-1\right).d_{X_{Pol}}^{BS}\right) \end{array}$$

(13) $$\begin{array}{c} {\vec{a}}_{l^{\prime }k^{\prime }}{\left(\varphi ,\vartheta \right)}_{V}=\left[\begin{array}{c} \chi_{l^{\prime }k^{\prime }}^{\vartheta }\chi_{l^{\prime }k^{\prime }}^{\varphi } \end{array}\right]\exp\left(js_{l^{\prime }k^{\prime }}\left(l^{\prime }k^{\prime }-1\right).d_{V}^{UE}\right) \end{array}$$

(14) $$\begin{array}{c} {\vec{a}}_{l^{\prime }k^{\prime }}{\left(\varphi ,\vartheta \right)}_{H}=\left[\begin{array}{c} \chi_{l^{\prime }k^{\prime }}^{\vartheta }\chi_{l^{\prime }k^{\prime }}^{\varphi } \end{array}\right]\exp\left(js_{l^{\prime }k^{\prime }}\left(l^{\prime }k^{\prime }-1\right).d_{H}^{UE}\right) \end{array}$$

(15) $$\begin{array}{c} {\vec{a}}_{l^{\prime }k^{\prime }}{\left(\varphi ,\vartheta \right)}_{V/H}=\left[\begin{array}{c} \chi_{l^{\prime }k^{\prime }}^{\vartheta }\\ \chi_{l^{\prime }k^{\prime }}^{\varphi } \end{array}\right]\exp\left(js_{l^{\prime }k^{\prime }}\left(l^{\prime }k^{\prime }-1\right).d_{V/H}^{UE}\right) \end{array}$$

where (.) is the scalar product, $d_{X_{Pol}}^{BS}$ is the location of $lk_{th}$ antenna element at the TX, and $\left(d_{V}^{UE},d_{H}^{UE},d_{V/H}^{UE}\right)$ are the locations of $l^{\prime }k_{th}^{\prime }$ antenna element at the RX. Also $s_{lk}$ and $s_{l^{\prime }k^{\prime }}$ are the TX and RX wave vectors, respectively, where $s_{lk}=\omega \frac{2\pi }{\lambda_{lk}}\hat{y}$ and $s_{l^{\prime }k^{\prime }}=\frac{2\pi }{\lambda_{l^{\prime }k^{\prime }}}\hat{y^{\prime }}$ with λ is the wavelength of the carrier frequency and outgoing wave $\hat{y}$ is equal to (sinθcosϕsinθsinϕcosθ) and incoming wave $\hat{y^{\prime }}$ is equal to $\left(\sin\vartheta \cos\varphi_{n}\sin\vartheta \sin\varphi \cos\vartheta \right)$ which are the directions of wave propagation in the response of the TX and RX antennas, respectively. Elevation angles (θ,ϑ) are defined between $90^{\circ }$ and $-35^{\circ }$, azimuth angles (ϕ,φ) are defined between $30^{\circ }$ and $150^{\circ }$ at the TX and RX, respectively. ω is the beam weight with $lk_{th}$ antenna element.

### 2.2. 3-D Beam Pattern

To make it easier for the reader to understand the use of the 3-D beam pattern technique in practice, we are going to investigate the use of 3-D antenna beam pattern, where different TX signals are fed to all the antenna elements with corresponding weights. However, we are interested in the channel between the TX antenna elements and RX antenna elements. The combination patterns for the transmit antenna array in dB are as follow [18]:

(16) $$\begin{array}{c} A_{E}^{BS}\left(\phi ,\theta ,\theta^{tilt}\right)=G_{E,max}^{BS}-\min\left(-\left[A_{H}^{BS}\left(\phi \right)+A_{V}^{BS}\left(\theta ,\theta^{tilt}\right)\right],20\right), \end{array}$$

where

(17) $$A_{V}^{BS}\left(\theta ,\theta^{tilt}\right)=-\min\left[12{\left(\frac{\theta -\theta^{tilt}}{\theta_{3dB}}\right)}^{2},20\right]dB,$$

(18) $$A_{H}^{BS}\left(\phi \right)=-\min\left[12{\left(\frac{\phi }{\phi_{3dB}}\right)}^{2},20\right]dB.$$

Therefore, the horizontal and vertical patterns at the TX can be approximated as

(19) $$\begin{array}{c} gt_{V}^{BS}\left(\theta ,\theta^{tilt}\right)=-12{\left(\frac{\theta -\theta^{tilt}}{\theta_{3dB}}\right)}^{2}dB, \end{array}$$

(20) $$\begin{array}{c} gt_{H}^{BS}\left(\phi \right)=-12{\left(\frac{\phi }{\phi_{3dB}}\right)}^{2}dB. \end{array}$$

where, $G_{E,max}^{BS}$ is the gain of the radiation element, which are assumed to be 7 dBi at the TX for each antenna elements. $A_{H}^{BS}\left(\phi \right)$ and $A_{V}^{BS}\left(\theta ,\theta^{tilt}\right)$ are horizontal and vertical patterns at the TX. The individual antenna radiation pattern at the UE $gr_{V}^{UE}\left(\vartheta ,\vartheta^{tilt}\right)$ and $gr_{H}^{UE}\left(\varphi \right)$ is taken to be 0 dB.

Given the antenna configuration in Section 2.1.2 and the effective radio channel between TX and RX in Equation (11) for different antenna polarizations, can be hence be written as Equations (11.1)–(11.3).

## 3. A Practical Non-Stationary of 3-D Massive MIMO Channel Model

### 3.1. Generation of the Cluster/Channel in System Level

**Figure 2:**
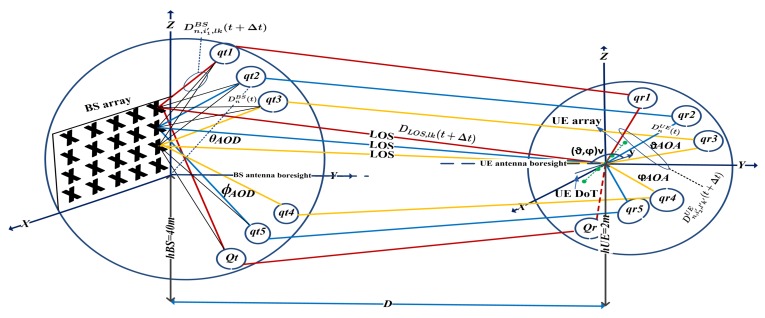
A more detailed of field experiments of the beam pattern using spherical coordinate system for non-stationary massive MIMO channel.

In this section, some important characteristics of the outdoor massive MIMO channel model for the far-field effects needs to be investigated, such as the distance between TX and RX, cluster generation and the appearance on the antenna array, movement direction of clusters and movement direction of antenna arrays. In Figure 2, all the details and dimensions of the non-stationary communication scenario for LOS component and NLOS components are shown. In addition, we implemented different effective scatters around the TX and RX. We also used a spherical model for dipole antenna elements at the TX and a spherical model for omnidirectional antenna elements at the RX to minimize cluster-dispersing under the beam pattern assumption. Regarding the non-stationary channel modeling, we need to distinguish the moving user in a cell, stationary of TX and random roadside environment (building, trees, cars, etc.). Therefore, to generate the channel coefficient, two parts must be modeled from the TX to the RX such as (a) toward the outdoor cluster at the TX, and (b) toward the receive antenna elements.

#### 3.1.1. Generating the Clusters at the Transmitter

Let us denote the $qt=\left\{1,\ldots ,Qt\right\}$, where Qt is equal to Cluster${}_{t}=Qt\left(t+\Delta t\right)$ as total clusters, in which ${i_{1}}_{th}$ path from ${lk}_{th}$ transmit antenna element is add up with time (t+Δt). In other words, the first path from ${\left(0,0\right)}_{th}$ antenna element with time *t* is add up to the nearest cluster. Additionally, the rest of the paths from ${lk}_{th}$ adjacent element calculating with distance $d^{BS}$ are add up with succeeding time instantaneously (Δt) to the same cluster.

#### 3.1.2. Toward the Receive Antenna Elements

$qt_{th}$ cluster will be observed to the $qr_{th}$ cluster at the RX. $qr=\left\{1,\ldots ,Qr\right\}$, where Qr is equal to Cluster${}_{t}=Qr\left(t+\Delta t\right)$ as total clusters, in which arrive to the ${l^{\prime }k^{\prime }}_{th}$ antenna element with time (t+Δt). Similarly, the first cluster with time *t* is arrived to the ${\left(0,0\right)}_{th}$ antenna element via ${i_{2}}_{th}$ path. Additionally, the rest of the clusters are arriving to adjacent element with succeeding time instantaneously (Δt) calculating with distance $d^{UE}$. As an example, cluster qt1 is observed to the cluster qr1, where qr1 is observed at the RX via ${i_{2}}_{th}$ path to the ${\left(0,0\right)}_{th}$ antenna element and so on.

To perform the evolution of outdoor channel coefficient, the clusters in the set of $\left(Qt(t+\Delta t)⋂Qr(t+\Delta t)\right)$ are generated based on the outdoor cluster evolution for outdoor communication. Based on the outdoor model, $\left(Cl_{n}^{out}\right)$ is the total number of clusters, where n=1,…,N cluster for outdoor communication can be expressed as

(21) $$\begin{array}{c} Cl_{n}^{out}=card\left(\overset{LK}{\underset{lk=1}{⋃}}\overset{L^{\prime }K^{\prime }}{\underset{l^{\prime }k^{\prime }=1}{⋃}}\left(Qt(t+\Delta t)⋂(Qr(t+\Delta t)\right)\right) \end{array}$$

where the operator card (.) denotes the cardinality of a set [8,14]. The operator card $Cl_{n}^{out}$, each cluster, say Cluster${}_{n}\left(n=1,\ldots ,Cl_{n}^{out}\right)$ is for outdoor models.

Based on the above analysis and the summary of key parameter definitions in Table 2, the massive MIMO channel matrix can be expressed as an $LK\times L^{\prime }K^{\prime }$ complex matrix $H\left(t,\Delta t,\tau \right)={\left[h_{lk,L^{\prime }k^{\prime }}\left(t+\Delta t,\tau \right)\right]}_{LK\times L^{\prime }K^{\prime }}$. The amplitude complex gains between the ${lk}_{th}$ antenna element at the LK transmit antenna, $l^{\prime }k_{th}^{\prime }$ antenna element at the $L^{\prime }K^{\prime }$ receive antenna and the delay τ, can be represented as

(22) $$H_{lk,l^{\prime }k^{\prime }}\left(t+\Delta t,\tau \right)=\sum_{n=1}^{Cl_{n}^{out}}h_{lk,l^{\prime }k^{\prime },n}\left(t+\Delta t\right)\delta \left(\tau -\tau_{n}\right)$$

1. if the Cluster${}_{n}$∈{Qt(t+Δt)⋂Qr(t+Δt)},
  (23) $$H_{n,lk,l^{\prime }k^{\prime }}\left(t+\Delta t,\tau \right)=\left(\begin{array}{c} \delta \left(n-1\right)\sqrt{\frac{G}{G+1}}\underset{LOS\;TX\;distance\;vector}{\underset{︸}{D_{LOS,i_{1},lk}^{BS}\left(t+\Delta t\right)}}\times \underset{LOS\;TX\;array\;response}{\underset{︸}{{\vec{a}\left(\phi ,\theta \right)}_{lk}}}\times \underset{LOS\;random\;phases}{\underset{︸}{\left[\begin{array}{cc} \exp(j\Phi_{LOS}^{v,v}) & 0\\ 0 & \exp(j\Phi_{n}^{h,h}) \end{array}\right]}}\times \\ \underset{LOS\;RX\;distance\;vector}{\underset{︸}{D_{LOS,i_{2},l^{\prime }k^{\prime }}^{UE}\left(t+\Delta t\right)}}\times \underset{LOS\;RX\;array\;response}{\underset{︸}{{\vec{a}}_{l^{\prime }k^{\prime }}\left(\varphi ,\vartheta \right)}}+\sqrt{\frac{A_{n}}{G+1}}\sum_{n=1}^{N}\underset{NLOS\;TX\;distance\;vector}{\underset{︸}{D_{n,i_{1},lk}^{BS}\left(t+\Delta t\right)}}\\ \times \underset{NLOS\;TX\;array\;response}{\underset{︸}{{\vec{a}\left(\phi ,\theta \right)}_{lk}}}\times \underset{NLOS\;random\;phases}{\underset{︸}{\left[\begin{array}{cc} \exp(j\Phi_{n}^{v,v}) & \sqrt{X^{h}}\exp\left(j\Phi_{n}^{v,h}\right)\\ \sqrt{X^{v}}\exp\left(j\Phi_{n}^{h,v}\right) & \exp(j\Phi_{n}^{h,h}) \end{array}\right]}}\times \\ \underset{NLOS\;RX\;distance\;vector}{\underset{︸}{D_{n,i_{2},l^{\prime }k^{\prime }}^{UE}\left(t+\Delta t\right)}}\times \underset{NLOS\;RX\;array\;response}{\underset{︸}{{\vec{a}}_{l^{\prime }k^{\prime }}\left(\varphi ,\vartheta \right)}} \end{array}\right),$$
  where *G* is the Rician factor and $A_{n}$ is the power of the $n_{th}$ cluster,
2. Otherwise, if Cluster${}_{n}\notin \left\{Qt\left(t+\Delta t\right)⋂Qr\left(t+\Delta t\right)\right\}$,
  (24) $$\begin{array}{c} H_{n,lk,l^{\prime }k^{\prime }}\left(t+\Delta t,\tau \right)=0 \end{array}$$

The calculation of complex gains for an outdoor model and description of the parameters in Equation (24) can be divided into NLOS components and LOS component both for TX and RX antenna arrays.

1. NLOS: The ${lk}_{th}$ transmit antenna element vector $\vec{a}{\left(\phi ,\theta \right)}_{lk}$ obtaining by Equation (12) in Section 2 and the vector between $n_{th}$ cluster via ${i_{1}}_{th}$ path $D_{n,i_{1},lk}^{BS}\left(t+\Delta t\right)$, and the vector between the $n_{th}$ cluster and the transmit antenna array $D_{n}^{BS}\left(t\right)$ at the TX can be given as
  (25) $$\begin{array}{c} D_{n,i_{1},lk}^{BS}\left(t+\Delta t\right)=D_{n}^{BS}\left(t\right){\left[\begin{array}{c} \sin\phi_{n,i_{1},lk}^{AAoD}\left(t+\Delta t\right),\cos\theta_{n,i_{1},lk}^{EAoD}\left(t+\Delta t\right)\\ \sin\phi_{n,i_{1},lk}^{AAoD}\left(t+\Delta t\right),\sin\theta_{n,i_{1},lk}^{EAoD}\left(t+\Delta t\right)\\ \cos\theta_{n,i_{1},lk}^{EAoD}\left(t+\Delta t\right) \end{array}\right]}^{T} \end{array}$$
  Similarly, the ${l^{\prime }k^{\prime }}_{th}$ receive antenna element vector $\vec{a}{\left(\varphi ,\vartheta \right)}_{l^{\prime }k^{\prime }}$ and the vector between $n_{th}$ cluster via ${i_{2}}_{th}$ path $D_{n,i_{2},l^{\prime }k^{\prime }}^{UE}\left(t+\Delta t\right)$, and the vector between the $n_{th}$ cluster and the receive antenna array $D_{n}^{UE}\left(t\right)$ at the RX can be presented as
  (26) $$\begin{array}{c} D_{n,i_{2},l^{\prime }k^{\prime }}^{UE}\left(t+\Delta t\right)=D_{n}^{UE}\left(t\right){\left[\begin{array}{c} \sin\varphi_{n,i_{2},l^{\prime }k^{\prime }}^{AAoA}\left(t+\Delta t\right),\cos\vartheta_{n,i_{2},l^{\prime }k^{\prime }}^{EAoA}\left(t+\Delta t\right)\\ \sin\varphi_{n,i_{2},l^{\prime }k^{\prime }}^{AAoA}\left(t+\Delta t\right),\sin\vartheta_{n,i_{2},l^{\prime }k^{\prime }}^{EAoA}\left(t+\Delta t\right)\\ \cos\vartheta_{n,i_{2},l^{\prime }k^{\prime }}^{EAoA}\left(t+\Delta t\right) \end{array}\right]}^{T}+D \end{array}$$
  Then, the four random initial phases for $n_{th}$ cluster are derived as
  (27) $$\begin{array}{c} \left[\begin{array}{cc} \exp(j\Phi_{n}^{\left(v,v\right)}) & \sqrt{X^{h}}\exp\left(j\Phi_{n}^{\left(v,h\right)}\right)\\ \sqrt{X^{v}}\exp\left(j\Phi_{n}^{\left(h,v\right)}\right) & \exp(j\Phi_{n}^{\left(h,h\right)}) \end{array}\right] \end{array}$$
  where $\sqrt{X^{h}}$ and $\sqrt{X^{v}}$ are inverse crosses polarized for X${}_{Pol}$ (vv/hv and hh/vh) transmission, respectively. $j=\sqrt{-1}$ and $\Phi_{n}^{\left(v,v\right)}$, $\Phi_{n}^{\left(v,h\right)}$, $\Phi_{n}^{\left(h,v\right)}$, $\Phi_{n}^{\left(h,h\right)}$ are the four random initial phases of different polarization combination.
2. LOS: The ${lk}_{th}$ transmit antenna element vector $\vec{a}{\left(\phi ,\theta \right)}_{lk}$ and the vector between $LOS_{th}$ path $D_{LOS,i_{1},lk}^{BS}\left(t+\Delta t\right)$ can be expressed as
  (28) $$\begin{array}{c} D_{LOS,i_{1},lk}^{BS}\left(t+\Delta t\right)={\left[\begin{array}{c} \sin\phi_{LOS,i_{1},lk}^{AAoD}\left(t+\Delta t\right),\cos\theta_{LOS,i_{1},lk}^{EAoD}\left(t+\Delta t\right)\\ \sin\phi_{LOS,i_{1},lk}^{AAoD}\left(t+\Delta t\right),\sin\theta_{LOS,i_{1},lk}^{EAoD}\left(t+\Delta t\right)\\ \cos\theta_{LOS,i_{1},lk}^{EAoD}\left(t+\Delta t\right) \end{array}\right]}^{T} \end{array}$$
  Similarly, the ${l^{\prime }k^{\prime }}_{th}$ receive antenna element vector $\vec{a}{\left(\varphi ,\vartheta \right)}_{l^{\prime }k^{\prime }}$ and the vector between $LOS_{th}$ path $D_{LOS,i_{1},l^{\prime }k^{\prime }}^{UE}\left(t+\Delta t\right)$ can be presented as
  (29) $$\begin{array}{c} D_{LOS,i_{1},l^{\prime }k^{\prime }}^{UE}\left(t+\Delta t\right)={\left[\begin{array}{c} \sin\varphi_{LOS,i_{1},l^{\prime }k^{\prime }}^{AAoA}\left(t+\Delta t\right),\cos\vartheta_{LOS,i_{1},l^{\prime }k^{\prime }}^{EAoA}\left(t+\Delta t\right)\\ \sin\varphi_{LOS,i_{1},l^{\prime }k^{\prime }}^{AAoA}\left(t+\Delta t\right),\sin\vartheta_{LOS,i_{1},l^{\prime }k^{\prime }}^{EAoA}\left(t+\Delta t\right)\\ \cos\vartheta_{LOS,i_{1},l^{\prime }k^{\prime }}^{EAoA}\left(t+\Delta t\right) \end{array}\right]}^{T}+D \end{array}$$
  Then, the two random initial phases for outdoor $LOS_{th}$ cluster is derived as
  (30) $$\begin{array}{c} \left[\begin{array}{cc} \exp(j\Phi_{LOS}^{v,v}) & 0\\ 0 & \exp(j\Phi_{LOS}^{h,h}) \end{array}\right] \end{array}$$
  where $\Phi_{LOS}^{\left(v,v\right)}$, $\Phi_{LOS}^{\left(h,h\right)}$ are two random initial phases of different polarization combination.

The generation of the cluster for each antenna element of the non-stationary process is based on the array-time evolution of clusters and can be divided into generation of cluster for each antenna according to Equation (22), and the second part is an updated model of geometrical relationships with respect to the movements of the receiver and clusters determines all parameters for each cluster. In the first part, the contribution of the models not only the appearance and disappearance of the cluster on antenna arrays, but also non-stationary behaviors of the clusters on the time axis. The array-time cluster evolution is describing based on the algorithm where assumed per meter as the number of $clusters_{n}$ and the initial cluster sets of the first transmit and receive antennas based on the Section 2, at the initial time *t* , then, the cluster sets for $\left(Qt(t+\Delta t)⋂Qr(t+\Delta t)\right)$ for both TX and RX recursively generate the cluster sets of the rest of antenna elements at the initial time instant *t* and Δ. Then, the cluster sets indices are reassigned from 1 to $cluster_{n}$.

### 3.2. Delay of the Clusters

The next step, the delay of the $n_{th}$ cluster for our proposed model, is based of the two components. The first component is according to the geometrical relationship between the transmitter-receiver arrays and cluster locations and the second component is the delay of the virtual link between two observed clusters at the TX and RX. Then, the delay of the $n_{th}$ cluster $\tau_{n}\left(t\right)$ can be formulated as [14]

(31) $$\begin{array}{c} \tau_{n}\left(t\right)=\frac{∥D_{n}^{BS}\left(t\right)∥+∥D_{n}^{UE}\left(t\right)∥}{c}+{\tilde{\tau }}_{n}\left(t\right) \end{array}$$

where the delay of the virtual link is ${\tilde{\tau }}_{n}\left(t\right)$ is according to the uniform distribution $U(D{/}_{c,\tau_{max}})$, and $\tau_{max}$ is the maximum delay equal to 1845 ns for NLOS [23], and c is the speed of light. Similarly, the delay of the $n_{th}$ cluster at t+Δt is expressed as the sum of the updated of the two-geometrical relationship and virtual link components between two observed clusters at the TX and RX.

(32) $$\begin{array}{c} \tau_{n}\left(t+\Delta t\right)=\frac{∥D_{n}^{BS}\left(t+\Delta t\right)∥+∥D_{n}^{UE}\left(t+\Delta t\right)∥}{c}+{\tilde{\tau }}_{n}\left(t+\Delta t\right) \end{array}$$

To evolute of the virtual link, its delay ${\tilde{\tau }}_{n}\left(t+\Delta t\right)$ is based on the first-order filtering algorithm as ${\tilde{\tau }}_{n}\left(t+\Delta t\right)=e^{-}{\left(\Delta /\zeta \right)}_{{\tilde{\tau }}_{n}\left(t\right)}+\left(1-e^{-}\left(\Delta /\zeta \right)\right)X$ where *X* is according to the uniform distribution $U(D{/}_{c,\tau_{max}})$, ζ is the coherence of a virtual link and scenarios [14].

### 3.3. Energy Transferring

Next, let’s consider the energy transferred between a transmitter and a receiver. A transmitter emits energy over time and the energy emitted per unit time is the power watts. If the transmitter is emitting radiation equally in all directions with power $P_{Ti}$, then the flux $F_{i}$ at a distance R from the transmitter is simply:

(33) $$\begin{array}{c} F_{i}=\frac{P_{Ti}}{4\pi R^{2}} \end{array}$$

## 4. Received Spatial Correlation

In practice, the channels between different antennas are often correlated, and therefore, the potential multi-antenna gains may not always be attainable [24]. We focus and analyze the spatial correlation of the receivers, including the effect of antenna polarization based on the practical multi-path wireless communication environment. We will then investigate and minimize such correlation at the RX by considering the AES to recognize the antenna element and its adjacent antenna elements from different polarization as well as changeable of the space $d^{UE}$. It is because of this purpose, in this paper, we developed the RSC based in [25]. For the received signals with the mean AAoA (φ) and EAoA (ϑ), the difference in their distance traveled is $d_{V}^{UE},d_{H}^{UE}$, and $d_{V/H}^{UE}$ for different polarizations. In addition, we are applying correlation for all possibilities antenna configuration for a given beam pattern. Let α and β denote the amplitude and phase antenna.

Therefore, their impulse response for V polarized elements can be represented as

(34) $$\begin{array}{c} h_{a}\left(\varphi_{V},\vartheta_{V}\right)=\alpha e^{j\beta }\sqrt{P(\varphi_{V},\vartheta_{V})} \end{array}$$

(35) $$\begin{array}{c} h_{b}\left(\varphi_{V},\vartheta_{V}\right)=\alpha e^{j\beta +\left(s_{l^{\prime }k^{\prime }}\left(l^{\prime }k^{\prime }-1\right).d_{V}^{UE}\right)}\sqrt{P(\varphi_{V},\vartheta_{V})} \end{array}$$

where $\sqrt{P(\varphi_{V},\vartheta_{V}})$ denote the PAS for V polarized.

In addition, their impulse response for H polarized elements can be given as

(36) $$\begin{array}{c} h_{a}\left(\varphi_{H},\vartheta_{H}\right)=\alpha e^{j\beta }\sqrt{P(\varphi_{H},\vartheta_{H})} \end{array}$$

(37) $$\begin{array}{c} h_{b}\left(\varphi_{H},\vartheta_{H}\right)=\alpha e^{j\beta +\left(s_{l^{\prime }k^{\prime }}\left(l^{\prime }k^{\prime }-1\right).d_{H}^{UE}\right)}\sqrt{P(\varphi_{H},\vartheta_{H})} \end{array}$$

where $\sqrt{P(\varphi_{H},\vartheta_{H}})$ denote the PAS for H polarized.

Furthermore, their impulse response for V/H polarized elements can be represented as

(38) $$\begin{array}{c} h_{a}\left(\varphi_{V/H},\vartheta_{V/H}\right)=\alpha e^{j\beta }\sqrt{P(\varphi_{V/H},\vartheta_{V/H})} \end{array}$$

(39) $$\begin{array}{c} h_{b}\left(\varphi_{V/H},\vartheta_{V/H}\right)=\alpha e^{j\beta +\left(s_{l^{\prime }k^{\prime }}\left(l^{\prime }k^{\prime }-1\right).d_{V/H}^{UE}\right)}\sqrt{P(\varphi_{V/H},\vartheta_{V/H})} \end{array}$$

where $\sqrt{P(\varphi_{V/H},\vartheta_{V/H}})$ denote the PAS for V/H polarized. Next, let us define a RSC function with the mean EAoA and AAoA of ϑ and φ in two antenna elements spaced apart by $d^{UE}+i$ as

(40) $$\rho_{d,\vartheta_{0},\varphi_{0}}^{V}=E\left\{h_{a}\left(\varphi_{V},\vartheta_{V}\right)\right\}\left\{h_{b}^{*}\left(\varphi_{V},\vartheta_{V}\right)\right\}=\int_{-\pi }^{\pi }\int_{\frac{-\pi }{2}}^{\frac{\pi }{2}}h_{a}\left(\varphi_{V},\vartheta_{V}\right)h_{b}^{*}\left(\varphi_{V},\vartheta_{V}\right)P\left(\varphi -\varphi_{0}\right)P\left(\vartheta -\vartheta_{0}\right)d_{\varphi }d_{\vartheta }$$

(41) $$\rho_{d,\vartheta_{0},\varphi_{0}}^{H}=E\left\{h_{a}\left(\varphi_{H},\vartheta_{H}\right)\right\}\left\{h_{b}^{*}\left(\varphi_{H},\vartheta_{H}\right)\right\}=\int_{-\pi }^{\pi }\int_{\frac{-\pi }{2}}^{\frac{\pi }{2}}h_{a}\left(\varphi_{H},\vartheta_{H}\right)h_{b}^{*}\left(\varphi_{H},\vartheta_{H}\right)P\left(\varphi -\varphi_{0}\right)P\left(\vartheta -\vartheta_{0}\right)d_{\varphi }d_{\vartheta }$$

(42) $$\begin{array}{c} \rho_{d,\vartheta_{0},\varphi_{0}}^{V/H}=E\left\{h_{a}\left(\varphi_{V/H},\vartheta_{V/H}\right)\right\}\left\{h_{b}^{*}\left(\varphi_{V/H},\vartheta_{V/H}\right)\right\}=\int_{-\pi }^{\pi }\int_{\frac{-\pi }{2}}^{\frac{\pi }{2}}h_{a}\left(\varphi_{V/H},\vartheta_{V/H}\right)h_{b}^{*}\left(\varphi_{V/H},\vartheta_{V/H}\right)\\ P\left(\varphi -\varphi_{0}\right)P\left(\vartheta -\vartheta_{0}\right)d_{\varphi }d_{\vartheta } \end{array}$$

**Figure 3:**
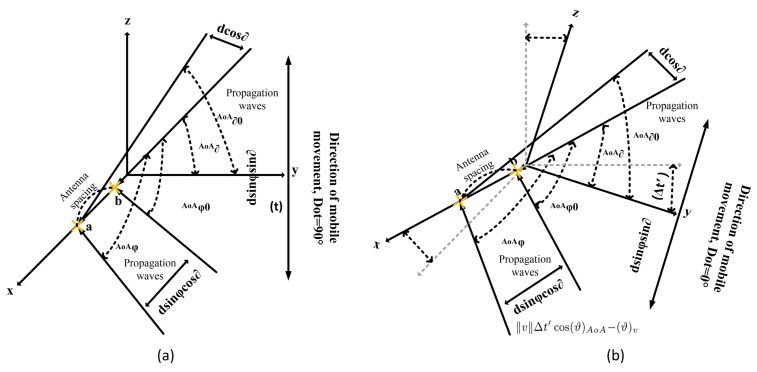
Phase difference between antenna elements for the varying (**a**) DoT = $90^{\circ }$ and (**b**) DoT = $0^{\circ }$.

However, in a non-stationary channel, EAoA, AAoA and angular spread (AS) are not equal to $0^{\circ }$. There is a time difference between $h_{a}\left(\varphi ,\vartheta \right)$ and $h_{b}\left(\varphi ,\vartheta \right)$ for V, H, and V/H polarized elements due to the Doppler Spread. In Figure 3 shows the phase difference between antenna elements under the DoT variations. This yields the following spatial correlation function for the SULA as

(43) $$\begin{array}{c} \rho_{d,\varphi_{0},\vartheta_{0}}^{V}\left(t+\Delta t\right)=E\left\{h_{a_{V}}\left(\vartheta ,\varphi \right)\right\}\left\{h_{b_{V}}^{*}\left(\vartheta ,\varphi \right)\right\}=\int_{-\pi }^{\pi }\int_{\frac{-\pi }{2}}^{\frac{\pi }{2}}h_{a}\left(\varphi_{V},\vartheta_{V}\right)h_{b}^{*}\left(\varphi_{V},\vartheta_{V}\right)P\left(\varphi -\varphi_{0}\right)P\left(\vartheta -\vartheta_{0}\right)d_{\varphi }d_{\vartheta }\\ \sum_{n=1}^{N}e^{(js_{l^{\prime }k^{\prime }}\left(l^{\prime }k^{\prime }-1\right).d_{V}^{UE}∥v∥\Delta t^{\prime }\cos{\left(\vartheta \right)}_{AoA}-{\left(\vartheta \right)}_{v})} \end{array}$$

(44) $$\begin{array}{c} \rho_{d,\varphi_{0},\vartheta_{0}}^{H}\left(t+\Delta t\right)=E\left\{h_{a_{H}}\left(\vartheta ,\varphi \right)\right\}\left\{h_{b_{H}}^{*}\left(\vartheta ,\varphi \right)\right\}=\int_{-\pi }^{\pi }\int_{\frac{-\pi }{2}}^{\frac{\pi }{2}}h_{a}\left(\varphi_{H},\vartheta_{H}\right)h_{b}^{*}\left(\varphi_{H},\vartheta_{H}\right)P\left(\varphi -\varphi_{0}\right)P\left(\vartheta -\vartheta_{0}\right)d_{\varphi }d_{\vartheta }\\ \sum_{n=1}^{N}e^{(js_{l^{\prime }k^{\prime }}\left(l^{\prime }k^{\prime }-1\right).d_{H}^{UE}∥v∥\Delta t^{\prime }\cos{\left(\vartheta \right)}_{AoA}-{\left(\vartheta \right)}_{v})} \end{array}$$

(45) $$\begin{array}{c} \rho_{d,\varphi_{0},\vartheta_{0}}^{V/H}\left(t+\Delta t\right)=E\left\{h_{a_{V/H}}\left(\vartheta ,\varphi \right)\right\}\left\{h_{b_{V/H}}^{*}\left(\vartheta ,\varphi \right)\right\}=\int_{-\pi }^{\pi }\int_{\frac{-\pi }{2}}^{\frac{\pi }{2}}h_{a}\left(\varphi_{V/H},\vartheta_{V/H}\right)h_{b}^{*}\left(\varphi_{V/H},\vartheta_{V/H}\right)\\ P\left(\varphi -\varphi_{0}\right)P\left(\vartheta -\vartheta_{0}\right)d_{\varphi }d_{\vartheta }\sum_{n=1}^{N}e^{(js_{l^{\prime }k^{\prime }}\left(l^{\prime }k^{\prime }-1\right).d_{V/H}^{UE}∥v∥\Delta t^{\prime }\cos{\left(\vartheta \right)}_{AoA}-{\left(\vartheta \right)}_{v})} \end{array}$$

where ${\left(\vartheta ,\varphi \right)}_{v}$ and ∥v∥ denote the DoT and mobile speed for different polarization, respectively. In case when DoT=$90^{\circ }$, the Equations (43)–(45) can be re-organized to

(46) $$\rho_{d,\varphi_{0},\vartheta_{0}}^{V}\left(t+\Delta t\right)=\sum_{n=1}^{N}e^{(js_{l^{\prime }k^{\prime }}\left(l^{\prime }k^{\prime }-1\right).d_{V}^{UE}∥v∥\sin{\left(\vartheta \right)}_{AoA})}$$

(47) $$\rho_{d,\varphi_{0},\vartheta_{0}}^{H}\left(t+\Delta t\right)=\sum_{n=1}^{N}e^{(js_{l^{\prime }k^{\prime }}\left(l^{\prime }k^{\prime }-1\right).d_{H}^{UE}∥v∥\sin{\left(\vartheta \right)}_{AoA})}$$

(48) $$\rho_{d,\varphi_{0},\vartheta_{0}}^{V/H}\left(t+\Delta t\right)=\sum_{n=1}^{N}e^{(js_{l^{\prime }k^{\prime }}\left(l^{\prime }k^{\prime }-1\right).d_{V/H}^{UE}∥v∥\sin{\left(\vartheta \right)}_{AoA})}$$

According to the above procedure of channel modeling, generating the realistic channel coefficient to evaluate the three aforementioned scenarios V, H and V/H polarized antenna elements in (11.1)–(11.3) can be extend to Equations (49)–(51) when DoT = $0^{\circ }$ and Equations (52)–(54) when DoT = $90^{\circ }$.

(49) $$h_{lk,l^{\prime }k^{\prime },n}^{V}\left(t+\Delta t,\tau \right)=\left(\begin{array}{c} \delta \left(n-1\right)\sqrt{\frac{G}{G+1}}D_{LOS,i_{1},lk}^{BS}\left(t+\Delta t\right)\times \vec{a}{\left(\phi ,\theta \right)}_{lk}\times \left[\begin{array}{cc} \exp(j\Phi_{LOS}^{v,v}) & 0\\ 0 & \exp(j\Phi_{LOS}^{h,h}) \end{array}\right]\times D_{LOS,i_{1},l^{\prime }k^{\prime }}^{UE}\left(t+\Delta t\right)\\ \times \exp(js_{l^{\prime }k^{\prime }}\left(l^{\prime }k^{\prime }-1\right).d_{V}^{UE}∥v∥\Delta t^{\prime }\cos{\left(\vartheta \right)}_{AoA}-{\left(\vartheta \right)}_{v})+\sqrt{\frac{A_{n}}{G+1}}\sum_{n=1}^{N}D_{n,i_{1},lk}^{BS}\left(t+\Delta t\right)\times \vec{a}{\left(\phi ,\theta \right)}_{lk}\times \\ \left[\begin{array}{cc} \exp(j\Phi_{n}^{\left(v,v\right)}) & \sqrt{X^{h}}\exp\left(j\Phi_{n}^{\left(v,h\right)}\right)\\ \sqrt{X^{v}}\exp\left(j\Phi_{n}^{\left(h,v\right)}\right) & \exp(j\Phi_{n}^{\left(h,h\right)}) \end{array}\right]\times D_{n,i_{2},l^{\prime }k^{\prime }}^{UE}\left(t+\Delta t\right)\times \\ \exp(js_{l^{\prime }k^{\prime }}\left(l^{\prime }k^{\prime }-1\right).d_{V}^{UE}∥v∥\Delta t^{\prime }\cos{\left(\vartheta \right)}_{AoA}-{\left(\vartheta \right)}_{v}) \end{array}\right)$$

(50) $$h_{lk,l^{\prime }k^{\prime },n}^{H}\left(t+\Delta t,\tau \right)=\left(\begin{array}{c} \delta \left(n-1\right)\sqrt{\frac{G}{G+1}}D_{LOS,i_{1},lk}^{BS}\left(t+\Delta t\right)\times \vec{a}{\left(\phi ,\theta \right)}_{lk}\times \left[\begin{array}{cc} \exp(j\Phi_{LOS}^{v,v}) & 0\\ 0 & \exp(j\Phi_{LOS}^{h,h}) \end{array}\right]\times D_{LOS,i_{1},l^{\prime }k^{\prime }}^{UE}\left(t+\Delta t\right)\\ \times \exp(js_{l^{\prime }k^{\prime }}\left(l^{\prime }k^{\prime }-1\right).d_{H}^{UE}∥v∥\Delta t^{\prime }\cos{\left(\vartheta \right)}_{AoA}-{\left(\vartheta \right)}_{v})+\sqrt{\frac{A_{n}}{G+1}}\sum_{n=1}^{N}D_{n,i_{1},lk}^{BS}\left(t+\Delta t\right)\times \vec{a}{\left(\phi ,\theta \right)}_{lk}\times \\ \left[\begin{array}{cc} \exp(j\Phi_{n}^{\left(v,v\right)}) & \sqrt{X^{h}}\exp\left(j\Phi_{n}^{\left(v,h\right)}\right)\\ \sqrt{X^{v}}\exp\left(j\Phi_{n}^{\left(h,v\right)}\right) & \exp(j\Phi_{n}^{\left(h,h\right)}) \end{array}\right]\times D_{n,i_{2},l^{\prime }k^{\prime }}^{UE}\left(t+\Delta t\right)\times \\ \exp(js_{l^{\prime }k^{\prime }}\left(l^{\prime }k^{\prime }-1\right).d_{H}^{UE}∥v∥\Delta t^{\prime }\cos{\left(\vartheta \right)}_{AoA}-{\left(\vartheta \right)}_{v}) \end{array}\right)$$

(51) $$h_{lk,l^{\prime }k^{\prime },n}^{V/H}\left(t+\Delta t,\tau \right)=\left(\begin{array}{c} \delta \left(n-1\right)\sqrt{\frac{G}{G+1}}D_{LOS,i_{1},lk}^{BS}\left(t+\Delta t\right)\times \vec{a}{\left(\phi ,\theta \right)}_{lk}\times \left[\begin{array}{cc} \exp(j\Phi_{LOS}^{v,v}) & 0\\ 0 & \exp(j\Phi_{LOS}^{h,h}) \end{array}\right]\times D_{LOS,i_{1},l^{\prime }k^{\prime }}^{UE}\left(t+\Delta t\right)\\ \times \exp(js_{l^{\prime }k^{\prime }}\left(l^{\prime }k^{\prime }-1\right).d_{V/H}^{UE}∥v∥\Delta t^{\prime }\cos{\left(\vartheta \right)}_{AoA}-{\left(\vartheta ,\varphi \right)}_{v})+\sqrt{\frac{A_{n}}{G+1}}\sum_{n=1}^{N}D_{n,i_{1},lk}^{BS}\left(t+\Delta t\right)\times \vec{a}{\left(\phi ,\theta \right)}_{lk}\times \\ \left[\begin{array}{cc} \exp(j\Phi_{n}^{\left(v,v\right)}) & \sqrt{X^{h}}\exp\left(j\Phi_{n}^{\left(v,h\right)}\right)\\ \sqrt{X^{v}}\exp\left(j\Phi_{n}^{\left(h,v\right)}\right) & \exp(j\Phi_{n}^{\left(h,h\right)}) \end{array}\right]\times D_{n,i_{2},l^{\prime }k^{\prime }}^{UE}\left(t+\Delta t\right)\times \\ \exp(js_{l^{\prime }k^{\prime }}\left(l^{\prime }k^{\prime }-1\right).d_{V/H}^{UE}∥v∥\Delta t^{\prime }\cos{\left(\vartheta \right)}_{AoA}-{\left(\vartheta \right)}_{v}) \end{array}\right)$$

Similarly, when DoT = $90^{\circ }$, the realistic channel coefficients are presented as

(52) $$h_{lk,l^{\prime }k^{\prime },n}^{V}\left(t+\Delta t,\tau \right)=\left(\begin{array}{c} \delta \left(n-1\right)\sqrt{\frac{G}{G+1}}D_{LOS,i_{1},lk}^{BS}\left(t+\Delta t\right)\times \vec{a}{\left(\phi ,\theta \right)}_{lk}\times \left[\begin{array}{cc} \exp(j\Phi_{LOS}^{v,v}) & 0\\ 0 & \exp(j\Phi_{LOS}^{h,h}) \end{array}\right]\times D_{LOS,i_{1},l^{\prime }k^{\prime \prime }}^{UE}\left(t+\Delta t\right)\\ \times \exp(js_{l^{\prime }k^{\prime }}\left(l^{\prime }k^{\prime }-1\right).d_{V}^{UE}∥v∥\sin{\left(\vartheta \right)}_{AoA})+\sqrt{\frac{A_{n}}{G+1}}\sum_{n=1}^{N}D_{n,i_{1},lk}^{BS}\left(t+\Delta t\right)\times \vec{a}{\left(\phi ,\theta \right)}_{lk}\times \\ \left[\begin{array}{cc} \exp(j\Phi_{n}^{\left(v,v\right)}) & \sqrt{X^{h}}\exp\left(j\Phi_{n}^{\left(v,h\right)}\right)\\ \sqrt{X^{v}}\exp\left(j\Phi_{n}^{\left(h,v\right)}\right) & \exp(j\Phi_{n}^{\left(h,h\right)}) \end{array}\right]\times D_{n,i_{2},l^{\prime }k^{\prime }}^{UE}\left(t+\Delta t\right)\times \exp(js_{l^{\prime }k^{\prime }}\left(l^{\prime }k^{\prime }-1\right).d_{V}^{UE}∥v∥\sin{\left(\vartheta \right)}_{AoA}) \end{array}\right)$$

(53) $$h_{lk,l^{\prime }k^{\prime },n}^{H}\left(t+\Delta t,\tau \right)=\left(\begin{array}{c} \delta \left(n-1\right)\sqrt{\frac{G}{G+1}}D_{LOS,i_{1},lk}^{BS}\left(t+\Delta t\right)\times \vec{a}{\left(\phi ,\theta \right)}_{lk}\times \left[\begin{array}{cc} \exp(j\Phi_{LOS}^{v,v}) & 0\\ 0 & \exp(j\Phi_{LOS}^{h,h}) \end{array}\right]\times D_{LOS,i_{1},l^{\prime }k^{\prime }}^{UE}\left(t+\Delta t\right)\\ \times \exp(js_{l^{\prime }k^{\prime }}\left(l^{\prime }k^{\prime }-1\right).d_{H}^{UE}∥v∥\sin{\left(\vartheta \right)}_{AoA})+\sqrt{\frac{A_{n}}{G+1}}\sum_{n=1}^{N}D_{n,i_{1},lk}^{BS}\left(t+\Delta t\right)\times \vec{a}{\left(\phi ,\theta \right)}_{lk}\times \\ \left[\begin{array}{cc} \exp(j\Phi_{n}^{\left(v,v\right)}) & \sqrt{X^{h}}\exp\left(j\Phi_{n}^{\left(v,h\right)}\right)\\ \sqrt{X^{v}}\exp\left(j\Phi_{n}^{\left(h,v\right)}\right) & \exp(j\Phi_{n}^{\left(h,h\right)}) \end{array}\right]\times D_{n,i_{2},l^{\prime }k^{\prime }}^{UE}\left(t+\Delta t\right)\times \exp(js_{l^{\prime }k^{\prime }}\left(l^{\prime }k^{\prime }-1\right).d_{H}^{UE}∥v∥\sin{\left(\vartheta \right)}_{AoA}) \end{array}\right)$$

(54) $$h_{lk,l^{\prime }k^{\prime },n}^{V/H}\left(t+\Delta t,\tau \right)=\left(\begin{array}{c} \delta \left(n-1\right)\sqrt{\frac{G}{G+1}}D_{LOS,i_{1},lk}^{BS}\left(t+\Delta t\right)\times \vec{a}{\left(\phi ,\theta \right)}_{lk}\times \left[\begin{array}{cc} \exp(j\Phi_{LOS}^{v,v}) & 0\\ 0 & \exp(j\Phi_{LOS}^{h,h}) \end{array}\right]\times D_{LOS,i_{1},l^{\prime }k^{\prime }}^{UE}\left(t+\Delta t\right)\\ \times \exp(js_{l^{\prime }k^{\prime }}\left(l^{\prime }k^{\prime }-1\right).d_{V/H}^{UE}∥v∥\sin{\left(\vartheta \right)}_{AoA})+\sqrt{\frac{A_{n}}{G+1}}\sum_{n=1}^{N}D_{n,i_{1},lk}^{BS}\left(t+\Delta t\right)\times \vec{a}{\left(\phi ,\theta \right)}_{lk}\times \\ \left[\begin{array}{cc} \exp(j\Phi_{n}^{\left(v,v\right)}) & \sqrt{X^{h}}\exp\left(j\Phi_{n}^{\left(v,h\right)}\right)\\ \sqrt{X^{v}}\exp\left(j\Phi_{n}^{\left(h,v\right)}\right) & \exp(j\Phi_{n}^{\left(h,h\right)}) \end{array}\right]\times D_{n,i_{2},l^{\prime }k^{\prime }}^{UE}\left(t+\Delta t\right)\times \exp(js_{l^{\prime }k^{\prime }}\left(l^{\prime }k^{\prime }-1\right).d_{V/H}^{UE}∥v∥\sin{\left(\vartheta \right)}_{AoA}) \end{array}\right)$$

According to the movement of the user from the transmitter, the power flux drops as the square of the distance. Another side, In the far-field situation the fluxes is smaller than the nearby shell that is computed by power per unit because the same energy will be spread over a larger area.

## 5. Experimental Results and Discussions

**Figure 4:**
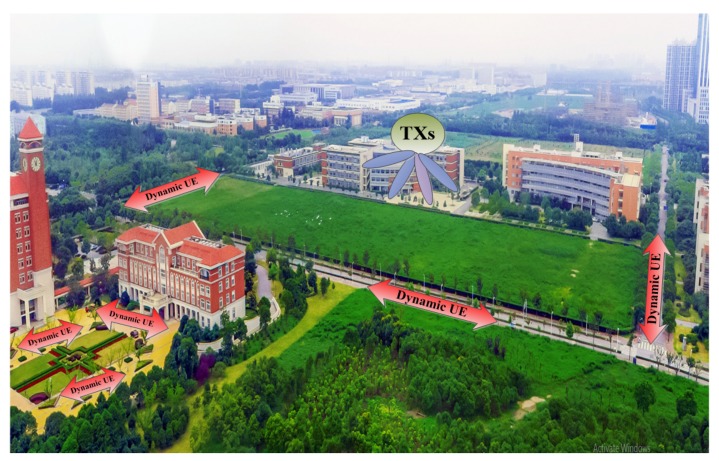
Overview of the measurement area at the campus of Shanghai Jiao Tong University, China. A spaced rectangular antenna array with 16 cross polarized patch antenna elements at the TX was placed on the rooftop of the Biomedical building during their respective measurement campaigns and a spaced uniform linear array as a user was moved around the international buildings acting as multiple-antenna user.

In this section, the statistical properties of the proposed model and simulation model are evaluated and analyzed. Then, the proposed simulation channel model is further validated by measurements. As shown in Figure 4, an investigation has been made then to study such full characterization through field experimentation on 3-D massive MIMO systems at Shanghai Jiao Tong University (SJTU) campus network. Sixteen cross-dipole elements with $+/-45^{\circ }$ slant polarization, and a movable user platform from the national instrument (NI) USRP-2943R with omnidirectional elements were implemented at the TX and RX, respectively. As shown in Figure 1, ${\left(90/90\right)}^{\circ }$ is a V polarization, ${\left(0/0\right)}^{\circ }$ is H polarization and ${\left(0/90\right)}^{\circ }$ is V/H polarization of omnidirectional antenna elements at the RX, and $+/-45^{\circ }$ dipole antenna elements at the TX are configured. The height above the BS is about 40 m, and the reception antenna’s height is 2.0 m with multiple receive antennas.

To verify our proposed channel models, we use MU-MIMO measurement data to compare the non-stationary of a measured MU-MIMO channel with that of standard channel models such as IMT-A and WINNER II. The result in investigating the performance of the proposed model and comparing them with the theoretical model. In order to understand the compression part easily, we denoted our proposed model as (E3-D) and the theoretical model as (C3-D). To highlight the impact of the statistical properties, we first extracted the theoretical model as a reference and then, and then an investigation of C3-D was carried out. Later, to get a more quantitative understanding of how our proposed model (E3-D) would perform in the real propagation and measured data on the proposed model, we turned our attention to the RSC and ECC in the three measured scenarios, corresponding to their DoTs, where different polarizations were used at the TX and RX. It shows that the spatial correlation and capacity of the theoretical model and practical result. The model was based on EAoA (ϑ), AAoA (φ), distance between the corresponding cluster to the antenna elements and arrays, elements spacing $\left(d^{BS},d^{UE}\right)$, antenna polarization, user movements and non-stationary of clusters. It shows that the practical model provides a good approximation to the theoretical model especially at dull polarization.

First, to understand the impact of the channel, we focused on the channel variation’s case of the distance traveled as well as considering the antenna polarization match/mismatching between TX and RX, while we formulated and analyzed data based on Section 3 and Section 4. The impact of the channel on the theoretical model and the corresponding proposed model of channel models affect the trends of the antenna polarization and spatial correlation properties considerably of the antenna elements. Second, RSC on ECC for correlation properties has been investigated, considering that the ASE had increased the spacing between elements. Consequently, as shown in the following figs, we are only going to investigate the performance of channel modeling with the effect of V, H, and V/H antenna polarization over E3-D channel, comparing with the C3-D model. This observation also was from the dynamic property of clusters on the array axis which may observe different sets of clusters with different ϑ and φ to at least one antenna element including the adjacent ones in the E3-D model. In addition, they demonstrate that proposed model provides a good approximation to the statistical properties of the theoretical model.

**Figure 5:**
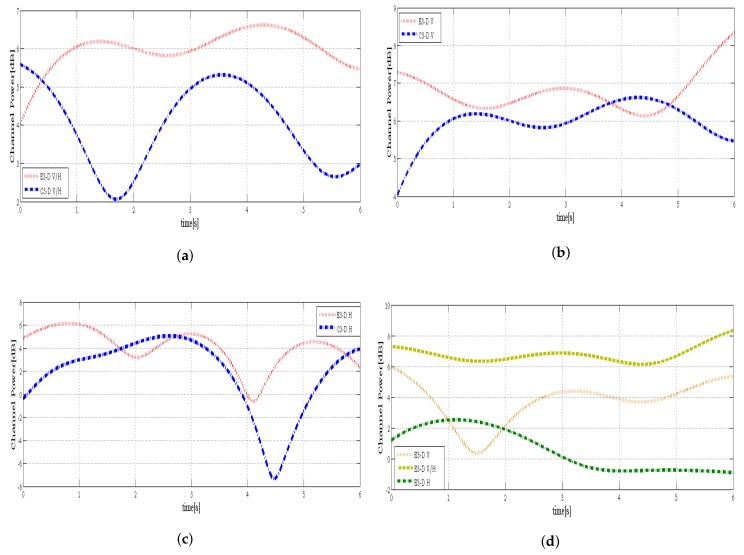
Performance comparison between E3-D and C3-D models based on different configurations, (**a**). V/H, (**b**). V, (**c**). H, receiving signals from TX with cross antenna elements polarization toward RX, in case there are polarization matching between TX and RX. And (**d**), in case there are polarization mismatching between TX and RX, the channel variations are based on the user movement with different DoT = $0^{\circ }$ and DoT = $90^{\circ }$. It is illustrated that the channel has been changed when the distance is increasing and also, the signal orientations do not match with the antenna polarization at the RX. Therefore, variation in polarization causes changes in the received signal level due to the inability of the antenna to receive such polarization changes.

As shown in Figure 5, the compression of the C3-D and E3-D massive MIMO model in different antenna configurations shows that the antenna polarization incorporated in the propagated channel cannot be ignored in channel modeling, especially when the distance between the TX and RX increasing. In addition, when DoT is not fixed along the axis but is switching from $0^{\circ }$ to $90^{\circ }$ or vice versa, it is also can be a polarization changing of antenna elements. This variation occurs due to the dynamic condition of the user between wave directions in relation to antenna polarization at the RX from time to time. For more details in Figure 5a–c show the massive MIMO channel variation based on H, V and V/H polarization where the waves are forced to lie in the same direction as the RX and the maximum pickup results when the RX antenna is oriented in the same direction as the TX antenna. The result also has shown that, in the C3-D, the conventional massive MIMO covering only an azimuth angle for far-field effect with providing equal AODs and AoAs, while in the E3-D model, the elevation angle has been considered with various phase excitation and different AoDs and AoAs for each antenna element. On the other side is shown in Figure 5d, when the cross-polarization antenna is configured at the TX, different polarization are assembled at the RX. The excellent agreement between the proposed MU-MIMO model and the measurement data demonstrates the utility of our MU-MIMO channel models.

**Figure 6:**
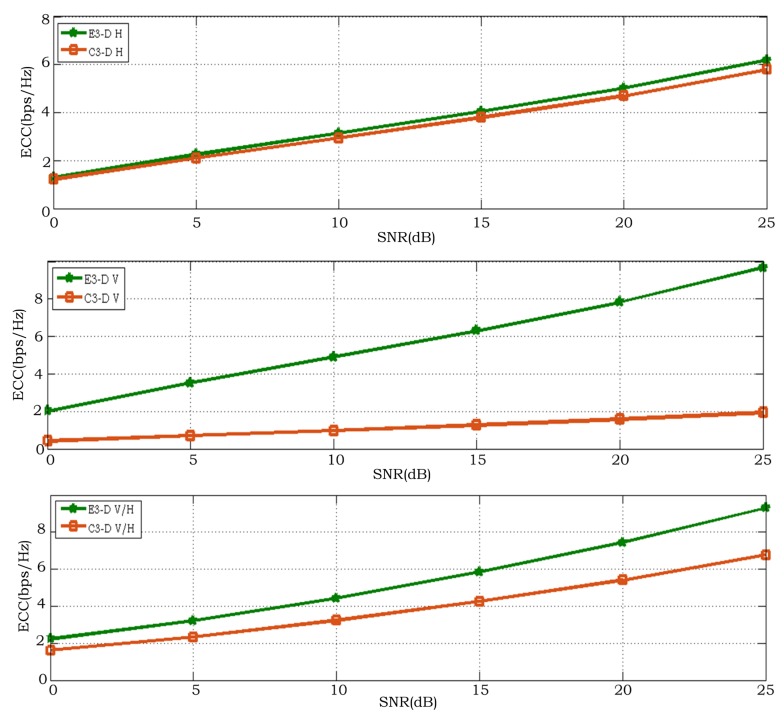
E3-D and C3-D compression capacity versus SNR for different polarization.

Moreover, the ECC of the period of clusters at the receivers on the antenna array, including the effect of antenna polarization is illustrated in Figure 6. Here, we only considered the cross-polarization antenna at the TX with different polarizations (V, H, and V/H) at the RX based on DoT = $0^{\circ }$. Then, we also investigated the influence of the channel noise on the achievable spectral efficiency. We defined the SNR for a non-stationary user as the ratio of the desired signal power to the noise power according to [26]

(55) $$SNR_{\left(V\right)}=\frac{{\left(h_{V}\right)}^{T}h_{V}}{\sigma_{n}^{2}}$$

(56) $$SNR_{\left(H\right)}=\frac{{\left(h_{H}\right)}^{T}h_{H}}{\sigma_{n}^{2}}$$

(57) $$SNR_{\left(V/H\right)}=\frac{{\left(h_{V/H}\right)}^{T}h_{V/H}}{\sigma_{n}^{2}}$$

where $\sigma_{n}^{2}$ denotes the power of channel noise and ${\left(h_{V}\right)}^{T}h_{V}$, ${\left(h_{H}\right)}^{T}h_{H}$, and ${\left(h_{V/H}\right)}^{T}h_{V/H}$ are the correlated channel matrix of complex path gains for V, H, and V/H polarized antennas, respectively. Therefore, a correlated capacity channel using the aforementioned expressions for different polarizations are derived as

(58) $$C_{\left(V\right)}=log_{2}\left[det\left(I+SNR_{V}\right)\right]bps/Hz$$

(59) $$C_{\left(H\right)}=log_{2}\left[det\left(I+SNR_{H}\right)\right]bps/Hz$$

(60) $$C_{\left(V/H\right)}=log_{2}\left[det\left(I+SNR_{V/H}\right)\right]bps/Hz$$

where, *det(.)* denotes the determinant and *I* is the identity matrix. From the figure, it can be observed that the ECC does not only depend on the antenna dimension, but it also has a close relation to the antenna configuration of the antenna array. Similarly, in V and V/H polarized antennas, the capacity is much higher than H polarization, because the elevation angle in the E3-D model has a higher degree of freedom and the main DoT is perpendicular to the receiver array.

In other words, the capacity of antenna elements polarization is lower than the others, causing the horizontally-receiving signals at an azimuth angle with a lower degree of freedom in elevation angle. This phenomenon occurs when the channel is linear to antenna elements, and the EAoA is constant for all elements. Therefore, based on the distance D and increased dimension of the antenna at the RX, spherical waves are emitted in a spherical shape and consider as a plane wavefront. However, the beam patterns with different phase excitation toward different DoTs, contributes various correlation weights for rays related to the Qt clusters. Thus, providing different EAoAs for each antenna element that can be adapted to the improve the received SNR which in turn to increases the accuracy of the channel model.

**Figure 7:**
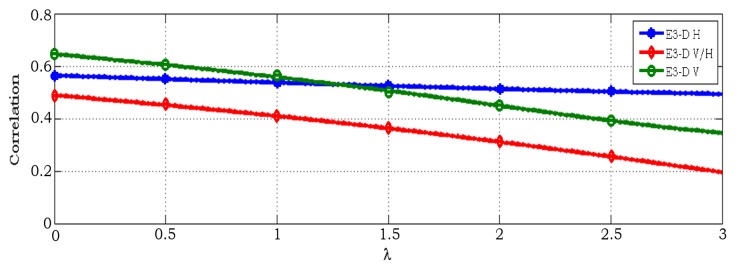
Correlation versus receiving antenna spacing on the beam pattern.

**Figure 8:**
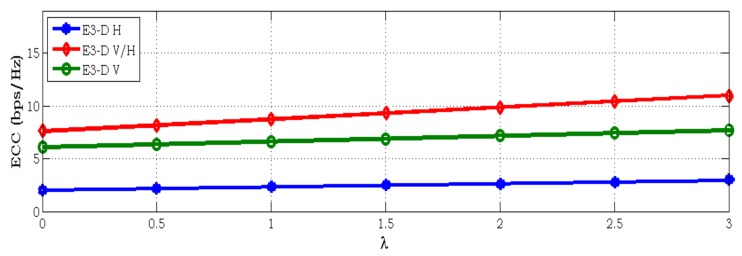
ECC versus receiving antenna spacing on the beam pattern.

Furthermore, an investigation of antenna correlation ρ versus space has been considered for the beam pattern as shown in Figure 7. It is shown that the correlation varies as the function of ρ(φ,ϑ) changes with $d^{UE}$ on ϑ and φ. Intuitively, the distance between two inter-elements is at the maximum, while the correlation remains minimal. The figure illustrated that the channel has less correlation in V and V/H configurations when the space between antenna elements increases. Also, H polarization varies slightly based on antenna space, because in H polarization the natural distance of elements from this kind of configuration is sufficient. Therefore, when space increase, the correlation result remains constant along the array axis. Finally, we investigated the effect of ECC versus antenna space at different polarization levels. Similar experimental observations can be found in Figure 8, where the capacity results also depending on the space between the antenna elements at the RX. It means that as space increases, correlation decreases and then capacity increases as well. The capacity of V and V/H polarization increases gradually as $d^{UE}$ while the configuration with H polarization is almost constant.

In summary, simulation results show that the H polarization antenna is not suitable for a 3-D model if, (A) uses plane wave front for the far-field effects, (B) the height of the user increases gradually, and (C) the dimension of antenna increases. In addition, the V polarization channel is sufficient when the only vertical angle is considered, and the spaces between antenna elements are enough for any reduction of correlation. This phenomenon happens in E3-D models when vertical degrees have higher wave dispersion from the beam pattern. Considering the same result, it is observed that correlation is not affected the channel in H polarization since the distance is enough and V polarization has the highest correlation result among others. Based on the results, having a dual polarization (V/H) at the RX while considering cross-polarization at the TX is a better option to have more accurate channels that can have at least one polarization at a certain time and direction of the user.

## 6. Conclusions

In the far-field condition, the key characteristics of massive MIMO channels such as the elevation angle and phase excitation were not captured by conventional massive MIMO in 3-D. In this model, we characterized the performance of the RSC for spaced linear antenna array by considering the scatters which were distributed around the TX and RX. Prior to this, an investigation considering AES was made to show the difference between 3-D beam pattern and 3-D conventional massive MIMO for V, H, and V/H polarizations at the RX. In addition, the result has shown an excellent relation between the derived theoretical and the measured data for received spatial correlation. Furthermore, ergodic correlation capacity was computed as a function of the received spatial correlation between elements. Channel measurement has been studied to examine the potential increase in capacity which can be achieved through a different spatial channel. Our research work in practice provided useful insights about the impact of antenna patterns and channel coefficients on the achievable capacity rate. It also confirmed the potential of elevated 3-D beam pattern in the enhancement of system performance. Furthermore, the channel models could be considered in both theoretical and practical guidance for the establishment of a more purposeful massive MIMO measurement in the future in any kind of antenna pattern.

## Figures and Tables

**Table 1:** Parameters for measurements.

Parameters	Values
Frequency range	2.620–2.630 (GHz)
Duplexing	TDD and FDD
Channel coding	Turbo code
Channel bandwidth	10 (MHz)
FFT size	1024 sub-carriers
CP length	80, 72 normal
Total symbols	140 s
TX block configuration	50 Resource blocks
Modulation schemes	QPSK and QAM
Multiple access schemes	OFDM

**Table 2:** Summary of key parameter definitions.

Parameters	Values
(θ,ϕ), (ϑ,φ)	elevation and azimuth angles of the departure and arrival, respectively
$D_{n,i_{1},lk}^{BS}\left(t+\Delta t\right)$	distance vector between $n_{th}$ cluster and ${lk}_{th}$ transmit antenna element via ${i_{1}}_{th}$ path
$D_{n}^{BS}\left(t\right)$	distance vector between $n_{th}$ cluster and transmit antenna
$D_{n,i_{2},l^{\prime }k^{\prime }}^{UE}\left(t+\Delta t\right)$	distance vector between $n_{th}$ cluster and ${l^{\prime }k^{\prime }}_{th}$ receive antenna element via ${i_{2}}_{th}$ path
$D_{n}^{UE}\left(t\right)$	distance vector between $n_{th}$ cluster and receive antenna
$D_{LOS,i_{1},lk}^{BS}\left(t+\Delta t\right)$	distance vector between $LOS_{th}$ path and ${lk}_{th}$ transmit antenna element
$D_{LOS,i_{1},l^{\prime }k^{\prime }}^{UE}\left(t+\Delta t\right)$	distance vector between $LOS_{th}$ path and ${l^{\prime }k^{\prime }}_{th}$ receive antenna element
$\vec{a}{\left(\phi ,\theta \right)}_{lk},\vec{a}{\left(\varphi ,\vartheta \right)}_{l^{\prime }k^{\prime }}$	array response of the TX and RX, respectively
